# Aetiology of type 2 diabetes: an experimental medicine odyssey

**DOI:** 10.1007/s00125-025-06428-0

**Published:** 2025-05-02

**Authors:** Roy Taylor

**Affiliations:** Magnetic Resonance Centre, Translational and Clinical Research Institute, Campus for Ageing and Vitality, Newcastle University, Newcastle upon Tyne, UK

**Keywords:** Aetiology, Beta cell function, Liver fat, Pancreas fat, Pathophysiology, Personal fat threshold, Remission, Review, Twin cycles, Type 2 diabetes, Weight loss

## Abstract

This review describes a prolonged research endeavour to test the twin cycle hypothesis that type 2 diabetes is caused by fat-induced dysfunction of the liver and pancreas, guided by the happenstance of clinical practice. Testing of the personal fat threshold hypothesis, that individuals exhibit different levels of tolerance to intra-organ fat accumulation, is also described. Both hypotheses predict that type 2 diabetes is potentially reversible by weight loss. The results of the Counterpoint study supported the twin cycle hypothesis, leading to a second study which determined that short-duration diabetes was more likely to remit following the 10–15 kg weight loss. It also confirmed that remission was durable over 6 months on an isoenergetic, normal diet. Subsequently, it was shown that weight loss caused an immediate decrease of pancreas fat only in people with type 2 diabetes and also that postprandial incretin spikes after bariatric surgery had no role in normalising fasting plasma glucose. DiRECT, a 2 year randomised controlled study, demonstrated clinical utility, observing functional beta cell capacity to return almost to normal over 12 months. A small group of participants regained weight and redeveloped type 2 diabetes, allowing observation that the underlying pathophysiological mechanisms during onset of diabetes were as postulated by the twin cycle hypothesis. Major clinical benefit was demonstrated after a further 3 year follow-up in routine care, halving the incidence of serious adverse effect compared with the standard treatment control group. In answer to the question of whether individuals have a personal fat threshold for tolerance of fat, stepwise weight loss in people with type 2 diabetes and BMI in the range 21–27 kg/m^2^ resulted in remission in 70%, with a wide range of fat thresholds. Type 2 diabetes can be regarded as a condition of homogenous aetiology in genetically heterogenous individuals.

**Graphical Abstract:**

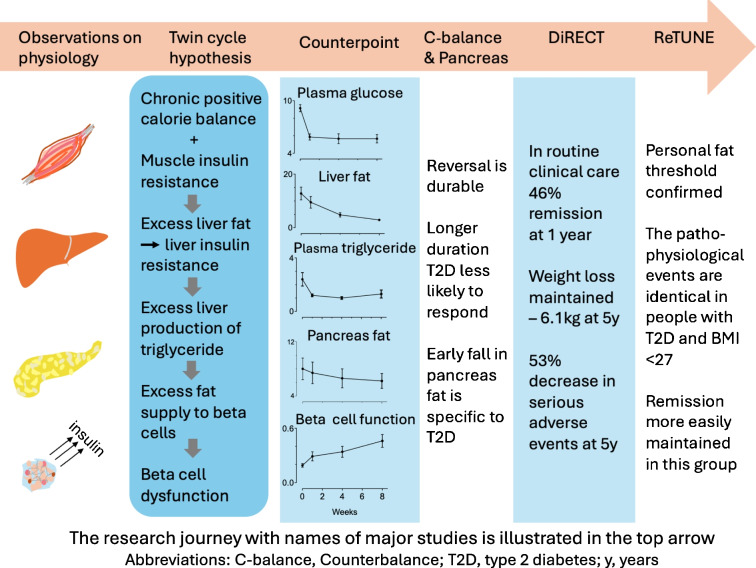

**Supplementary Information:**

The online version contains a slideset of the figures for download, which is available to authorised users available at 10.1007/s00125-025-06428-0.

## Introduction

Claude Bernard well deserves the title of Father of Experimental Medicine. He demonstrated that the processes within living tissues were explicable in scientific terms, challenging the universal belief that the functions of the body were controlled by a ‘vital force’. Claude Bernard pointed out that if an observation was thought about in the light of other knowledge then a hypothesis could be put forward to explain cause and effect. Such a hypothesis would predict the outcome of a change to the notional causative processes. His 1865 book ‘*An Introduction to Experimental Medicine*’ has timeless relevance and deserves to the read by all embarking upon a career in medical research today.

Each of his seminal discoveries was a research journey. Each started with a concept of the intended destination but with no map or guide book, just like a physical journey across uncharted territory.

## First steps: probing the cause of type 2 diabetes

In the 1980s the cause of type 2 diabetes was uncertain although contribution of both insulin resistance and beta cell defect were apparent [1]. I used biopsies of human adipose tissue and muscle to evaluate the cause of insulin resistance [2–4]. The possibility of a method kinder to research volunteers caused me to arrange a sabbatical with Jerry Shulman at Yale to learn magnetic resonance spectroscopic techniques [5]. We first established that the new method was accurate compared with direct biochemical measurement (*R*=0.95) [6]. Then we tackled a basic question: what is the time course of storage of meal-derived glucose as glycogen in normal health? We found that muscle glycogen peaked at a mean of 4 h after eating and steadily declined thereafter during a 7 h follow-up [7]. Provided muscle insulin sensitivity is normal, skeletal muscle acts as a dynamic buffer for incoming glucose, providing safe but temporary storage of this potentially toxic nutrient.

This raised the question of how glucose was handled in people with type 2 diabetes. Nottingham University had a suitable magnetic resonance scanner and expert physicists, and we collaborated and showed the increase in muscle glycogen to be minimal in type 2 diabetes [8]. Instead, a high proportion of ingested glucose would be converted to fat for safe storage (de novo lipogenesis), the only other available pathway. Even healthy young people with muscle insulin resistance convert around 16% of post-meal carbohydrate to fat [9]. These observations are important in understanding the chronic complications of type 2 diabetes because the sole product of de novo lipogenesis is palmitic acid (100% saturated fat). This is muscle insulin resistance in real life: silently promoting CVD via saturated fat, meal by meal. We subsequently showed that the dynamic buffer function of skeletal muscle is absent in type 2 diabetes [10]. No wonder that CVD is so closely associated with type 2 diabetes.

The conversion of glucose to fat happens only in the liver in humans. In an unrelated study, we found that all individuals with type 2 diabetes had hepatic steatosis on biopsy [11]. We followed this up and observed a close relationship between liver fat content and liver insulin resistance [12, 13]. Kitt Petersen and colleagues at Yale then demonstrated that this liver insulin resistance normalised after decreasing liver fat content by moderate weight loss [14]. This was a highly important observation, as fasting hyperglycaemia is caused by relatively unrestrained hepatic glucose production despite high fasting plasma insulin levels [15, 16]. Even after meals, the normal suppression of hepatic glucose output is only partial in type 2 diabetes, adding to the postprandial hyperglycaemia [13].

## Claude Bernard’s ‘thinking’ phase: planning the journey

By 2000, we needed a magnetic resonance centre in Newcastle to investigate the physiology of normal glucose handling in the body and the cause of type 2 diabetes. It took 4 years to raise the necessary €8 million, 2 years to build the centre, then several more years to assemble the team of brilliant physicists and others. While this was going on, the thinking was underway. How did the jigsaw of observations to date fit together to explain type 2 diabetes?

**Fig. 1:**
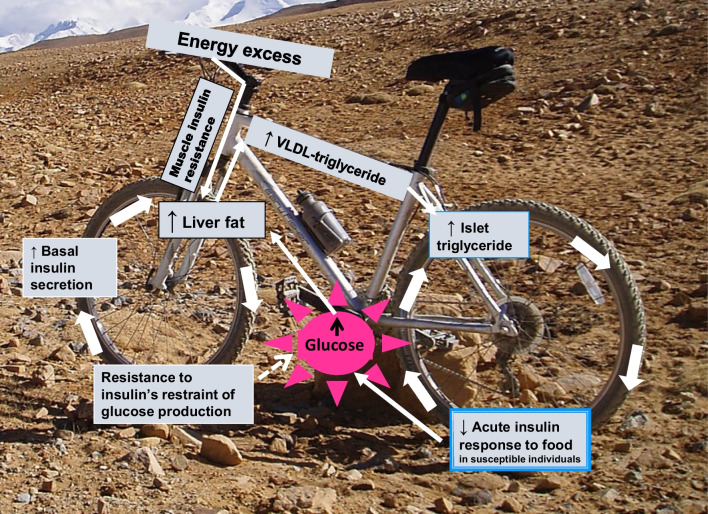
Every journey needs a vehicle, which for this research journey was the twin cycle hypothesis. According to this hypothesis, modest chronic positive energy balance leads to increased liver fat, and this is promoted by increased post-meal de novo lipogenesis resulting from muscle insulin resistance. The increased liver fat levels cause steadily increasing resistance to the normal insulin action of restraining hepatic glucose production. The resulting increase in fasting plasma glucose induces a compensatory increase in fasting plasma insulin. Insulin directly stimulates de novo lipogenesis, thus creating a vicious cycle in the liver. As liver fat levels increase, the normal rate of export of VLDL-triglyceride to the rest of the body increases. Especially when subcutaneous fat stores are relatively replete, triglyceride is oversupplied to ectopic sites, including the pancreas. Beta cells are exposed to excess toxic lipid intermediaries causing dysfunction in susceptible individuals, chronically suppressing the acute insulin response to food. The consequent increase in post-meal plasma glucose brings about prolonged hyperinsulinaemia, and the excess glucose and insulin further increase de novo lipogenesis. Hence the hypothesis postulates interacting vicious cycles leading to both hepatic insulin resistance and beta cell dysfunction initiated by the single environmental factor of chronic excess food intake. It predicts that type 2 diabetes is a reversible metabolic state and that glucose control would return to normal if the initiating factor was removed. These changes would be expected to reflect, in reverse, the events leading to the onset of type 2 diabetes. The figure is adapted, with permission, from [79]. This figure is available as part of a downloadable slideset

A starting point was that in the long term, slight surplus of food appeared likely to explain the typical excess body weight at diagnosis of type 2 diabetes. That could lead to fat accumulation in the liver. Muscle insulin resistance, detectable in early life, is the first warning of susceptibility to type 2 diabetes [9, 17, 18]. The increased de novo lipogenesis and excess liver fat would cause the liver to become insulin resistant, with relatively uncontrolled glucose production provoking an increase in basal insulin production. As insulin stimulates de novo lipogenesis, a vicious cycle might be initiated, further increasing liver fat content, eventually leading to an increase in the continuous supply of fat from the liver to the rest of the body and increasing plasma triglyceride levels (the liver cycle; Fig. 1).

It had long been established that type 2 diabetes is driven by a combination of insulin resistance and subnormal beta cell function. Common diseases tend to have simple causation so could these factors be linked? The serendipity of clinical observation in the clinic struck at just the right time. A patient demanded to be rid of their type 2 diabetes. As the liver cycle appeared theoretically solid, I advised substantial weight loss to decrease liver fat content and possibly normalise fasting blood glucose. The problem of poor insulin secretion and post-meal hyperglycaemia would remain, I explained. This determined patient went away and achieved major weight loss, decreasing BMI to the level it had been in early adult life. To my surprise, not only did fasting glucose normalise but the OGTT returned entirely to normal. It looked as though the pancreas had recovered. How?

Triglyceride is metabolically inert when stored in subcutaneous tissues. If this storage depot becomes relatively replete, the fat would have to be deposited in ectopic sites including the pancreas. Could excess fat inhibit beta cell function? A decade previously, the pioneering work of the American physiologist Roger Unger had shown that long-term exposure of islets to saturated fat caused them to lose their normal response to an acute rise in glucose [19]. This happened only when islets were from animals genetically susceptible to type 2 diabetes if overfed and not from animals without this susceptibility. In humans, lipid infusion decreases beta cell function only in people genetically susceptible to type 2 diabetes [20, 21]. Suddenly, the pieces of the jigsaw fell into place (Fig. 1). Chronic exposure of beta cells to excess triglyceride might decrease the ability to increase insulin secretion in response to meals. Prolonged postprandial hyperglycaemia would provide more fuel for de novo lipogenesis, further increasing liver fat content. There would be a vicious cycle at the level of the pancreas as well as in the liver. These twin cycles would interact and be self-reinforcing (Fig. 1).

The whole point of developing a hypothesis is that it can be tested to destruction or confirmation. Each step of the hypothesis could be quantified as the physicists at the Newcastle Magnetic Resonance Centre were able to develop the new methods needed to measure intrahepatic and intrapancreatic fat. The latter accumulates equivalently in endocrine and exocrine cells [22, 23]. To test the hypothesis, it would be necessary, in Claude Bernard’s words, to ‘stimulate a change’. Removing the postulated cause (i.e. positive energy balance) would be predicted to normalise all the pathways, with around 15 kg weight loss being needed to decrease the level of fat inside the pancreas.

A reliable method was required to achieve this degree of weight loss consistently in research participants going about their normal lives. Listening to patients had taught me about the two main problems: first, nagging hunger; and second, the cumulative day-to-day burden of deciding how much of what food to eat. Decades-old research had established that hunger was less troublesome on < 1000 kcal/day diets (4184 kJ) [24]. Otherwise, complete nutrition could be provided on 700–800 kcal/day (2929–3347 kJ) to achieve a weight loss of 15 kg in 8 weeks. The second problem could be overcome if a commercially available liquid formula diet was used, with no burden of difficult decisions about what to eat and how much. Satiation would be helped by a high protein content (~ 25%) and constipation would be minimised by the addition of a daily large portion of non-starchy vegetables (such as salad foods). The finite duration of the weight loss period could be fitted around birthdays or other life events.

## Stimulating a change and repeating the observation

The critical research tool of an effective weight-loss method was ready to ‘stimulate a change’. The twin cycle hypothesis would be disproven if all the underlying processes and plasma glucose did not return to normal. Because the postulated effect size was large, the prior power calculation showed that only 12 people would be required to achieve a clear-cut positive or negative conclusion. The Counteracting Pancreatic inhibitiOn of INsulin secretion by Triglyceride (Counterpoint) study aimed to bring about 15 kg weight loss in people with type 2 diabetes in an 8 week period. Empirically, type 2 diabetes < 4 years from diagnosis was studied [25]. Oral glucose-lowering agents were stopped after the baseline tests on day 1, and the ~ 800 kcal/day (~ 3347 kJ/day) diet was started. A weight-matched control group without diabetes was also included in the study.

**Fig. 2:**
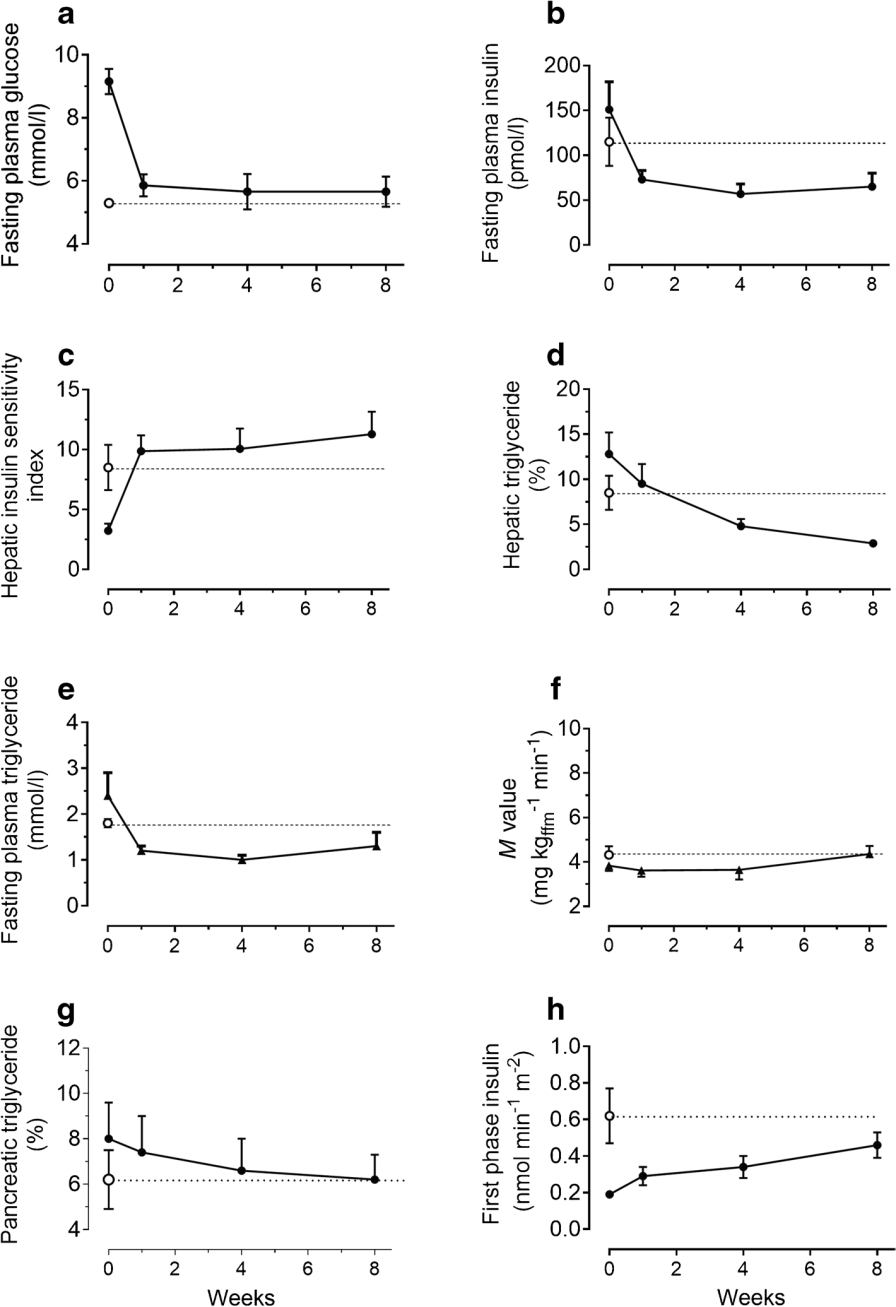
Testing the twin cycle hypothesis by the Counterpoint study. Data are shown for the test group (black circles) and for a matched normoglycaemic control group studied on a single occasion (white circles). Individual panels show baseline (day 0) and data after starting the ~ 800 kcal/day (~ 3347 kJ/day) diet plus stopping oral hypoglycaemic agents: (**a**) fasting plasma glucose; (**b**) fasting plasma insulin; (**c**) hepatic insulin sensitivity; (**d**) intra-hepatic triglyceride content as percentage of liver volume; (**e**) fasting plasma triglyceride; (**f**) whole-body insulin sensitivity determined by hyperinsulinaemic clamp; (**g**) intrapancreatic triglyceride content; and (**h**) first-phase insulin response to an acute (2.8 mmol/l) rise in plasma glucose. The figure is adapted, with permission, from [25]. ffm, fat-free mass. This figure is available as part of a downloadable slideset

The results of the Counterpoint study were electrifying. The mean weight loss over 8 weeks was 15.3 kg. Fasting plasma glucose levels normalised after only 7 days (Fig. 2a) and HbA_1c_ within 8 weeks (7.4 ± 0.3 to 6.0 ± 0.2%; 57 ± 2 to 42 ± 1 mmol/mol). Normalisation of all postulated physiological variables was exactly as predicted (Fig. 2b–h).

At baseline, in unremarkable type 2 diabetes, liver fat content was grossly elevated. It fell by one-third in the first 7 days, becoming similar to that of the control group. All triglyceride in the liver is intrahepatocellular but reflects the excess of the lipid intermediary diacylglycerol, which actually causes insulin resistance [26, 27]. The latter decreases very rapidly with negative energy balance [28], explaining normalisation of hepatic insulin sensitivity at 7 days. Fasting hepatic glucose production and plasma insulin levels normalise at the same time. The change in insulin sensitivity was entirely hepatic (Fig. 2c), with no significant change in muscle insulin resistance, confirming previous observations [14].

The elevated plasma triglyceride of type 2 diabetes has always been a puzzle but here was a way to completely normalise the 50% elevated level (Fig. 2e). It is worth pointing out that lipoprotein metabolism is very simple, despite being complicated in biochemistry textbooks. The liver secretes VLDL-triglyceride (VLDL-TG), a payload of triglyceride bound by a cholesterol membrane. The VLDL-TG particles gradually deliver triglyceride to all tissues and a range of progressively smaller particles are generated. In total, these make up plasma triglyceride. Plasma triglyceride concentration depends largely upon the rate of hepatic secretion of VLDL-TG. There are no other lipoproteins (HDL-cholesterol is not strictly a lipoprotein). Normalisation of fasting plasma triglyceride reflects decreased output of VLDL-TG by the liver [29]. This brings about continuous excess fatty acid delivery to all tissues, including the islets.

Hence, all tissues would experience a fall in triglyceride delivery, explaining the gradual decrease in intrapancreatic fat content (Fig. 2g). In step with this decrease over 8 weeks, the first-phase insulin response also returned towards normal. The early and complete correction of high glucose produced only a minor effect. This was the first time that beta cells in type 2 diabetes had been observed to wake up (Fig. 2h), permitting return of fasting and postprandial glucose control.

Hence, the Counterpoint study confirmed the physiological predictions of the twin cycle hypothesis. The scientific news was greeted with interest, but scepticism. The criticism ‘only a small study’ was frequently voiced, even though this test of a prior hypothesis was as determined by power calculation and observed highly statistically significant findings. In an age of extremely large studies, the importance of appropriately sized, precise tests of hypotheses is often forgotten. The larger a study needs to be to show a statistically significant effect, the less likely it is to be relevant to the patient in front of you. Nevertheless, it was clear that further studies were required.

## Advancing into the wilderness

What if the return to normal was not a true change of state but merely a starvation effect, with diabetes re-appearing on return to normal eating? This was tested in COUNTERacting BetA cell failure by Long term Action to Normalise Calorie intakE (Counterbalance), a larger study repeating the protocol of Counterpoint but with 6 months of normal isoenergetic eating after the weight loss phase [29]. Our research volunteers told us that the most difficult challenges of the rapid weight loss process was stopping the low-energy diet and adjusting to normal eating. Some described feeling panicky on going back into the kitchen, not certain what to cook or what to eat. We therefore devised a stepped return to normal eating, replacing one liquid meal at a time with normal food in specified quantities. Additionally, we tested whether normalisation was possible irrespective of duration of diagnosed type 2 diabetes.

**Fig. 3:**
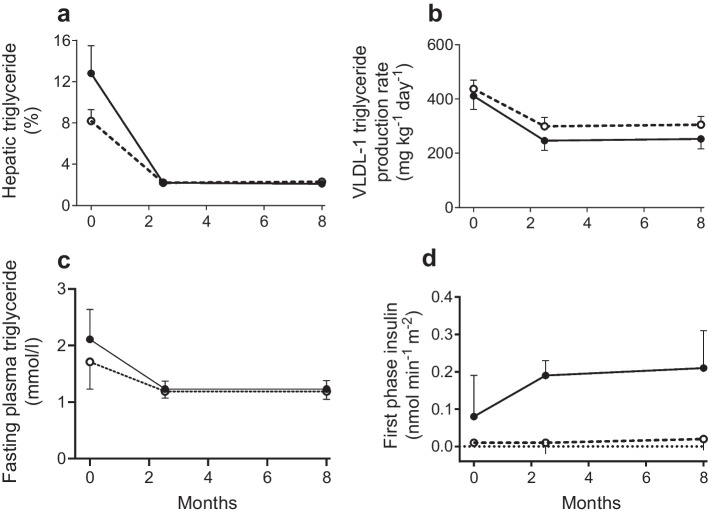
The Counterbalance study, determining whether post-weight-loss metabolic normality persisted after return to normal eating for 6 months. A group of participants with type 2 diabetes duration of up to 24 years was recruited to determine the effect of duration of type 2 diabetes. Data are shown for responders (black circles) who returned to a non-diabetic HbA_1c_ and for non-responders (white circles, dashed line) who did not. (**a**) Hepatic triglyceride levels were not significantly lower in non-responders (*p*=0.09) but post-weight-loss levels were almost identical. (**b**) The rate of liver production of triglyceride (measured as the dominant VLDL- 1 subfraction) decreased similarly in both groups. (**c**) Total plasma triglyceride levels decreased in both groups. (**d**) First-phase insulin response to a 2.8 mmol/l elevation of plasma glucose was notably poor at baseline in non-responders (with no response to weight loss) but the responders showed a good response to weight loss; this was maintained over the 8 months of the study. Data are plotted from [29]. This figure is available as part of a downloadable slideset

In Counterbalance, not everyone reversed their diabetes to normal, despite similar weight loss, clearly related to duration of previous diabetes. The group achieving remission had a mean diabetes duration of 3.8 years compared with 9.8 years in the non-responders. These data led to the adoption of a cut-off of less than 6 years’ duration in all subsequent studies on remission. This was not a binary matter but merely an expression of likelihood of achieving remission. Liver fat returned to normal (Fig. 3a) and direct measurement of VLDL-TG secretion by the liver confirmed that hepatic secretion explained the change in plasma triglyceride (Fig. 3b, c).

For those who did not return to non-diabetic HbA_1c_, it was striking that the liver cycle and hence plasma lipids returned to normal. The problem was in the beta cells (Fig. 3d): normalisation of beta cell function depended on duration of diabetes. Clinical practice has revealed considerable intra-individual variability in resilience to chronic exposure to excess fat. As extreme examples, two people with a diabetes duration of 23 years have since been observed to return to the non-diabetic state after weight loss. In the light of Roger Unger and co-workers’ observations [19], this beta cell resilience is likely to be genetically determined.

During Counterpoint we had been surprised to hear from the participants that they actually liked the diet, so in the Counterbalance study we explored this via independent psychologists. The very positive feedback was confirmed [30]: ‘the first week or so I was probably feeling hungry but after that, absolutely fine’; ‘it was fairly hard to start with but it got easier as the weeks went on, and then when I started getting a bit fitter and I could walk further and stand up and sit down and dig the garden, it’s great now. I feel great’. A common comment was ‘I feel 10 years younger’. The benefits were evident after the first week, and this was a powerful motivation to continue. Doctors frequently assume that adherence to such a diet is unfeasible, until they observe success in their first patient.

The 10 year cardiovascular risk score in Counterbalance normalised with remission (from 15.3± 3.4 to 5.8 ± 1.6%) [31]. The non-remitters had a worse risk score at baseline and improved but did not normalise (from 21.4 ± 2.9 to 15.0 ± 2.0%). Given the gloomy cardiovascular outlook typical of type 2 diabetes, this is very good news and a call to clinical action.

The rapidity of weight loss is important. Slow weight loss of sufficient degree can achieve the same effects but the success of rapid weight loss relates to human factors. We observed this very early on in the journey when a person with type 2 diabetes contacted me as soon as funding for Counterpoint was announced, wishing to participate. By the time regulatory approval had been granted 8 months later, she had lost 14 kg gradually merely by decreasing portion size and glucose tolerance had returned to normal.

Bariatric surgery produces impressive weight loss with rapid decrease in fasting plasma glucose. It became widely believed that this effect was due to spikes in incretin hormones after meals. However, the study on pancreatobiliary bypass, which popularised this, also disproved the concept. No food had been eaten until 7 days postoperatively. So normalisation of fasting plasma glucose at 7 days occurred before the pancreas had been exposed to any postprandial surge of incretins [32]. The identical effects of dietary energy restriction and bariatric surgery on glucose control has since been shown by direct comparison [33–35]. The incretin story can be laid to rest. Bariatric surgery certainly achieves more durable longer-term avoidance of weight regain but the mechanism of reversal of type 2 diabetes is nothing to do with incretin effects. Myths can so easily be widely accepted if methods sections of research papers are not read carefully.

The twin cycle hypothesis predicted that beta cell function would recover when an excess of intrapancreatic fat was removed. However, was change in pancreas fat merely reflective of generalised weight loss and not specific to type 2 diabetes? To answer this, the pancreas study examined intrapancreatic fat content after rapid weight loss in groups with and without type 2 diabetes. It confirmed that intrapancreatic fat was elevated at baseline and decreased only in those with type 2 diabetes. The observations were suggestive of raised intracellular lipid intermediaries and triglyceride levels that were rapidly decreased by weight loss [36, 37].

## Change of direction: could the research tool be applied to routine management of type 2 diabetes?

The original question regarding the aetiology of type 2 diabetes by now appeared beyond doubt but could the same method of rapid weight loss be used in everyday clinical practice to bring about remission of type 2 diabetes? From 2011 onwards, newspaper articles about Counterpoint had caused a flood of e-mail enquiries and a how-to-do-it website was set up. Then many e-mails reported outcomes of self-achieved reversal of type 2 diabetes. These were collated [38]. Return to normal blood glucose control was reported in 80% of those with > 20 kg weight loss, 63% of those losing 10–20 kg and 53% of those losing < 10 kg. However, anecdotal information was not sufficient. Evidence from RCTs was needed on how this research tool (the low-energy diet) could reasonably be applied in routine National Health Service (NHS) primary care. By this time Lean and colleagues had applied the low-energy liquid diet to obesity in primary care and we collaborated to design DiRECT [39].

Primary care nurses or dietitians were trained how to supervise the three phases of weight loss: low-energy diet; stepped return to normal eating; and then long-term avoidance of weight regain. Reviewers of the grant application were sceptical that recruitment would be possible. In practice, recruitment proved difficult to stop and we overshot the power calculation target (298 people recruited rather than 280) [40]. Even today, healthcare professionals tend to underestimate the number of people who detest having type 2 diabetes and will take any action likely to achieve return to health [41, 42].

At 1 year, 46% of the intervention group had achieved remission, with a mean weight loss of 10.0 kg [43]. In the control group managed by guidelines, 4% were in remission. It is challenging to maintain weight loss in the unchanged obesogenic environment and at 2 years the mean weight loss in the intervention group had slipped to 7.6 kg, with 36% still in remission [44]. However, those who achieved more than 15 kg weight loss had almost a 9/10 chance of being in remission [43, 44].

**Fig. 4:**
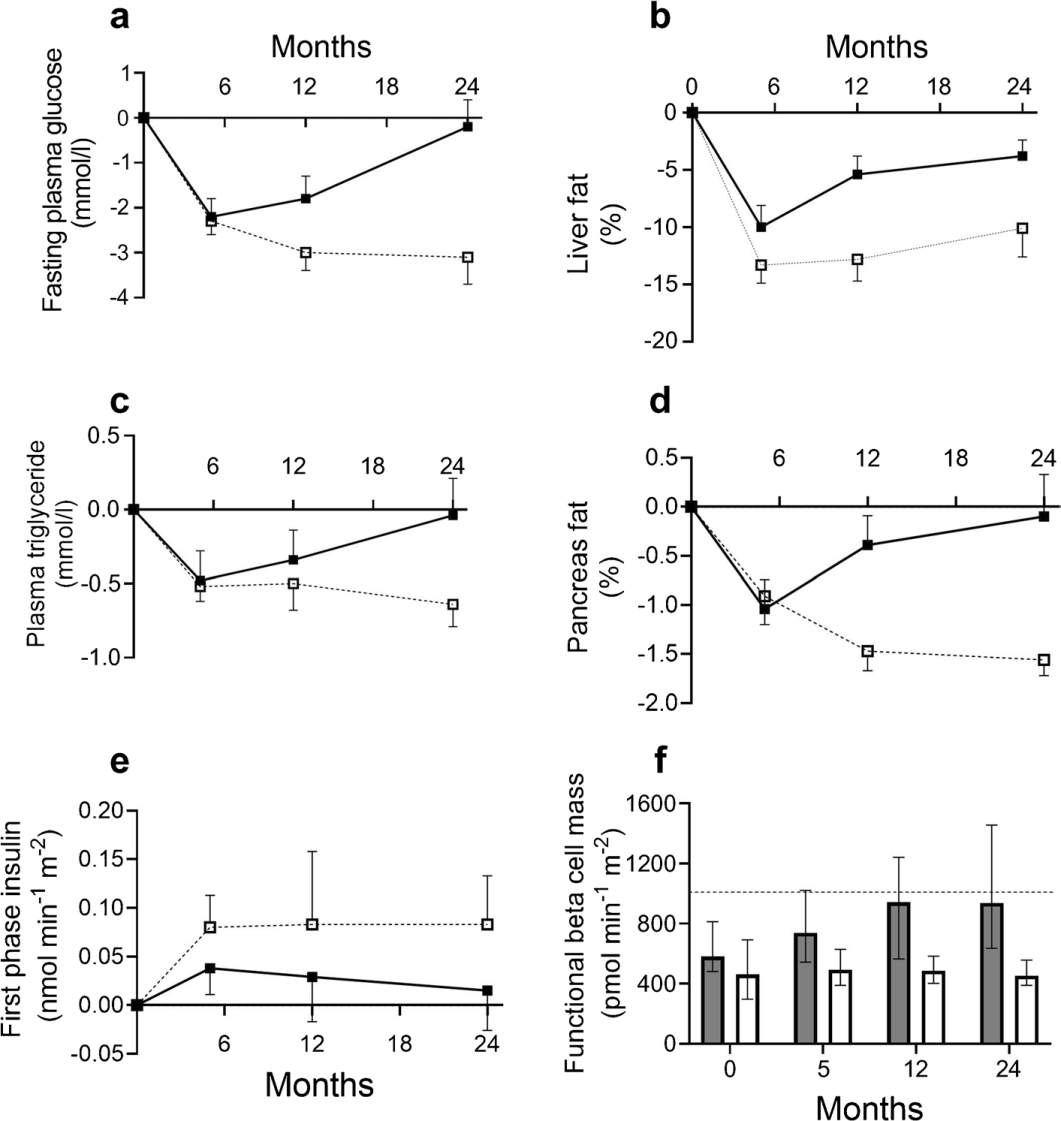
The DiRECT study provided the opportunity to confirm whether the pathophysiological processes of the twin cycle hypothesis would explain the forward direction leading to steady loss of glucose control. Data are shown for the group of 13 people who were in remission after weight loss at 5 months but gained sufficient weight over 2 years to allow type 2 diabetes to redevelop (black squares/white bars) compared with those who remained in remission (white squares/grey bars, dashed line). During the redevelopment of type 2 diabetes: (**a**) fasting glucose increased to baseline levels; (**b**) liver fat content gradually increased (shown as percentage of liver volume); (**c**) plasma triglyceride gradually increased; (**d**) the excess intrapancreatic fat gradually re-accumulated; (**e**) beta cell function recovered exactly as in those with continuing remission, becoming suppressed so that first-phase insulin response returned to baseline levels; and (**f**) the maximal beta cell capacity, reflecting functional beta cell mass in response to a 5.4 mmol/l elevation of plasma glucose plus an arginine bolus, returned almost to that of the normoglycaemic control group (horizontal dotted line). Data are plotted from [45, 47]. This figure is available as part of a downloadable slideset

A critical part of DiRECT was to observe the previously determined pathophysiological mechanisms over a longer period of time (Fig. 4) [45, 46]. The most remarkable observation was that functional beta mass for those in remission very slowly returned almost to 100% of non-diabetic levels, remaining constant thereafter (Fig. 4f) [47]. The first-phase insulin response improved after weight loss and remained constant provided weight regain was avoided. Clearly the beta cells had not been dead or apoptosed but merely their function had been suppressed by the excess fat. Removal of the cause allowed gradual recovery.

A small group of those in remission at 5 months regained more weight than they could tolerate and type 2 diabetes redeveloped [45]. We thus had a ringside seat to observe the underlying pathophysiological processes in real time as normal metabolism deteriorated to type 2 diabetes. Our earlier studies had shown the twin cycle hypothesis to be consistent with the reverse direction of change; now, we could track the causative processes forwards. For this group the weight regain had been sufficient to bring about a steady increase in liver, plasma and pancreas triglyceride. These individuals had already shown themselves to have beta cells susceptible to excess fat, and unsurprisingly first-phase insulin response decreased to the baseline type 2 diabetes level (Fig. 4e).

## Re-calibrating the destination

Given the widespread belief that gradual beta cell failure was inevitable in type 2 diabetes, it was important to track remission over a longer period in real-life clinical practice. Follow-up of the intervention group was funded by Diabetes UK for a total of 5 years. Mean weight was still 6.1 kg below baseline at 5 years with major benefit [48]. However, a weight loss of 6.1 kg was not enough to keep most people in remission. By 5 years, 26% of those in remission at 2 years had maintained this, by avoiding weight regain (mean 8.9 kg below baseline).

Critically, once the causative factor of excess intra-organ fat was removed there was no ongoing beta cell decline. The learned helplessness generated by observing inexorable progression in people with type 2 diabetes who remain too heavy can be dispelled. Beta cells do not deteriorate once protected from excess fat. The group mean weight increased steadily but weight regain frequently occurred during the intermittent and inevitable stresses of life (e.g. problems at work, family illness, financial issues) when focus on avoidance of excess food was lost. These human factors distracted the focus on careful eating. This illustrates that one of the commonest underlying factors for weight regain is not primarily biological but rather reflects those slings and arrows of everyday life. During DiRECT, the primary care nurses or dietitians provided pre-planned ‘rescue packages’ to help this problem. Around half of the intervention group required at least one rescue over 2 years [49].

Follow-up of DiRECT throughout was not designed to be optimal for weight control but rather to be low-cost (3-monthly visits to a primary care practice nurse or dietitian). The aim was to test an intervention which could feasibly be made by the UK NHS. During the extended follow-up, support was also less than ideal due to primary care staff turnover and pressure of time. The outcomes could have certainly been improved by more focused low-cost follow-up, perhaps using apps [50].

For people with type 2 diabetes, avoidance of illness caused by diabetes is of far more immediate concern than the numerical outcomes discussed in clinic. The number of serious adverse events requiring hospital treatment was more than halved in the intervention group at 5 years compared with the control group (by intention-to-treat analysis) [48]. This included eight weight-related cancers in the control group (the number expected for type 2 diabetes [51]) compared with only one in the intervention group [48]. The study length of DiRECT was too short to detect any change in cardiovascular events. However, longer-term outcomes of remission have been defined retrospectively by 12 year follow-up of substantial weight loss. The Look Ahead study achieved sufficient weight loss for 12% of participants to be in remission from type 2 diabetes at any time during the study. If remission was present for four or more annual follow-up visits, the cardiovascular risk was cut by 51% [52]. However, even if remission had been achieved for only one visit, risk was cut by 33%. The original observation from Counterbalance that 10 year cardiovascular risk score normalises during remission [31] has now been confirmed by real outcome data.

Establishing a definition of remission was important for both people with diabetes and researchers. Remission is the time-based extension of reversing the underlying processes causing type 2 diabetes. A joint statement from UK associations of primary care and specialist doctors defined it as two non-diabetic HbA_1c_s off all anti-hyperglycaemic agents over 6 months [53]. An international ADA, EASD and Diabetes UK consensus definition of remission reinforced and extended these criteria [54]. It clarified that weight control medication could be used during remission. The cut-off level of HbA_1c_ (< 48 mmol/mol [< 6.5%]) has since been challenged on the grounds that plasma glucose may be in the ‘pre-diabetes’ range. However, glucose is merely an associated indicator of risk during steady increase in plasma glucose levels and not the prime driver of macrovascular disease. Weight loss normalises liver fat and plasma lipids, resulting in both return to macrovascular health and remission of type 2 diabetes. The normalised plasma lipids following weight loss with non-diabetic HbA_1c_s are not associated with excess macrovascular risk [52]. This is completely different from the state of HbA_1c_ rising through the grey zone labelled as ‘pre-diabetes’ in which macrovascular disease is driven on by the abnormal plasma lipids. With respect to microvascular risk, the diagnostic level for diabetes was originally selected on grounds of the upwards inflection point on the risk graph [55]. Hence, the practical utility of the consensus definition of remission is strongly supported by outcome data on both micro- and macrovascular disease.

## Exploratory journey to autoroute

It cannot be assumed that good outcomes in research studies can be reproduced in routine clinical practice. So the NHS established a pilot study [56]. This low-cost 1 year programme was delivered by independent providers using health coaches rather than nurses or dietitians. Entry was solely via doctor referral. The only other doctor role was to agree withdrawal of oral glucose-lowering medications and one antihypertensive drug [57]. The latter was necessary to avoid postural hypotension on the low-energy, very-low-sodium diet. In the first 960 people completing a 1 year follow-up, a mean weight loss of 10.3 kg was achieved, with 35% having an HbA_1c_ < 48 mmol/mol (< 6.5%). The unnecessary prescription of metformin led to strict diagnosis of remission in 27%.

As a result of these clear-cut pilot study outcomes, NHS England has rolled out a nationwide ‘T2D Path to Remission Programme’. This is now operational in all regions, for people within 6 years of diagnosis. As the programme was based on trial evidence then available, it was restricted to those aged 25–65 years with BMI greater than 27 kg/m^2^. Discussion is underway about provision of longer-term support to maintain remission.

It must be emphasised that not everyone with type 2 diabetes wants to achieve freedom from the condition by taking charge of their health or wishes to undertake the weight loss. This is a treatment option that involves challenges. For those who wish to avoid medication, it offers long-term health provided weight regain is minimised. It is not possible to predict which patients will lose weight adequately. One solid observation from all our studies to date is that support from family and friends during and after weight loss is very valuable and should be sought in advance. However, if a spouse/partner is not supportive of weight loss it is unlikely to happen. Nonetheless, it is an option with remarkable long-term benefits that should be offered to all.

## Do drugs offer an easy route to major weight loss?

The new appetite suppressants such as terzepatide offer the potential for weight loss of similar extent [58]. It can confidently be anticipated that all the benefits demonstrated following major dietary weight loss will be achievable using these agents. Discontinuation of the drug is followed by rapid weight regain because there has been no element of learning how to eat for weight stability [59]. Possibly these agents could be used strategically, perhaps helping with episodes of weight regain after dietary weight loss. They are likely to play a major role in preparing for orthopaedic and other surgery, with the likely minimisation of infective or thrombotic complications and earlier discharge from hospital. Although pharmaceutical companies prefer to describe the agents as ‘incretin’ agents [58] there is no in vivo evidence that they affect beta cell function directly but rather remove fat-induced stress on beta cells [60]. They have potent effects in moving perceived appetite along the axis from ‘usually hungry’ to ‘not hungry’ or even nauseated, resulting in major weight loss. How these remarkable drugs will optimally be used in clinical practice requires careful study.

## Is type 2 diabetes aetiology the same in people with lower BMI?

The UK Prospective Diabetes Study (UKPDS) laid the basis for the modern understanding of type 2 diabetes although it is now largely forgotten that over one-third of people in UKPDS had a normal BMI at diagnosis [61]. This may seem surprising today, but at the time of recruitment (1970s to 1980s) only 7% of the background population were obese [62] and there appeared to be no link between type 2 diabetes and obesity [1, 63]. The risk of type 2 diabetes rises exponentially with increasing BMI so the association with obesity becomes very evident as a population becomes heavier [64, 65].

In Counterpoint and all subsequent studies, a 15 kg weight loss brought about identical changes in the underlying mechanisms over the full BMI range studied (27–45 kg/m^2^) [25, 29, 36, 46]. Remission was achieved with a decrease of around 3 BMI units across this range, so that individuals who dropped to BMI 42 kg/m^2^ or BMI 24 kg/m^2^ could be free of diabetes. However, it had to be considered whether the aetiological mechanisms might differ in people who appeared to carry no excess fat. A second hypothesis was postulated: that each person has an individual threshold beyond which they can no longer tolerate the fat burden inside the liver and pancreas, leading to type 2 diabetes. We used data on BMI distribution at baseline in the UKPDS to support publication of the personal fat threshold hypothesis [66].

**Fig. 5:**
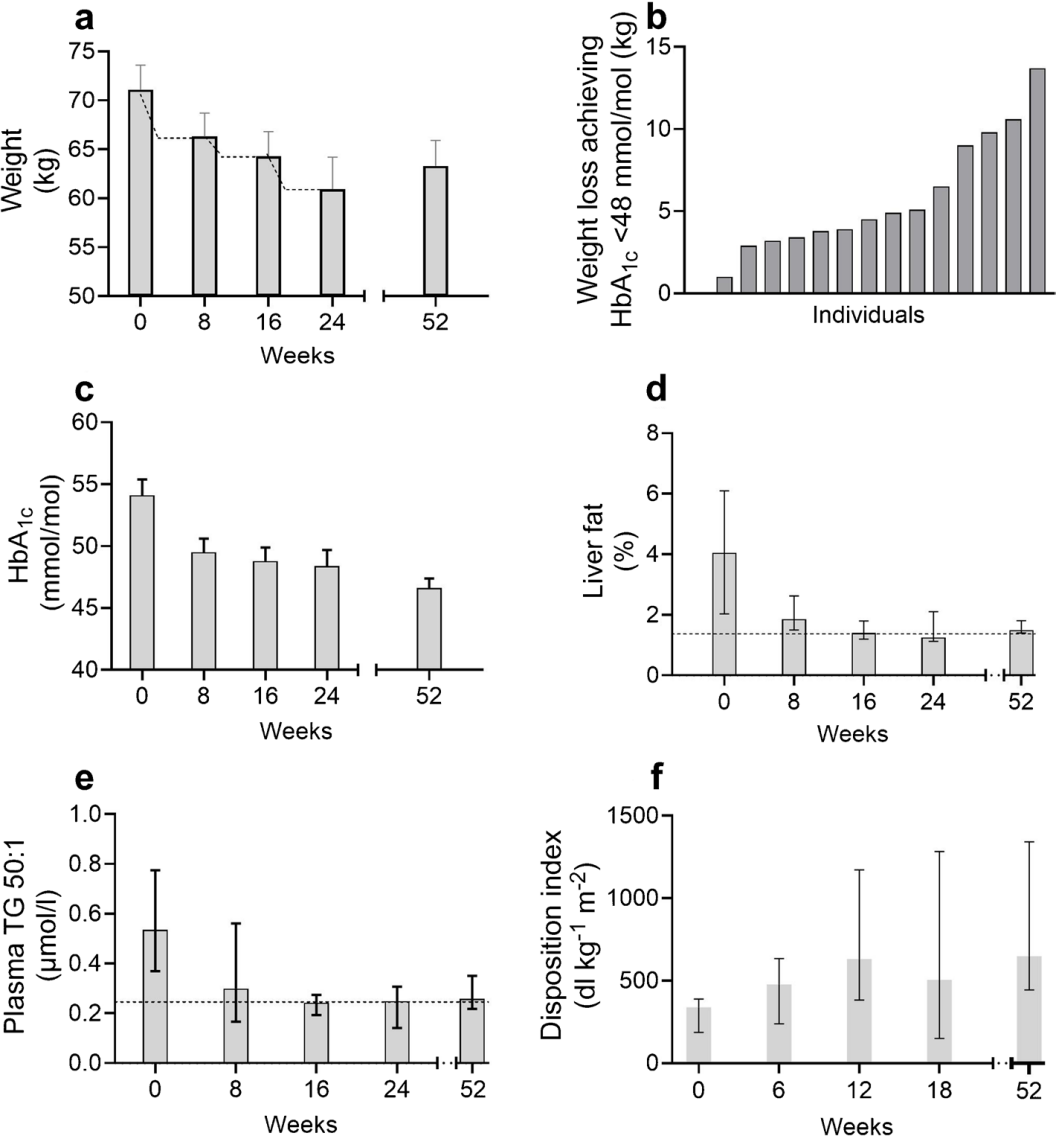
Testing the personal fat threshold hypothesis by the ReTUNE study. (**a**) Rapid weight loss of 5% was followed by a period of weight stability before re-testing. Only participants who had not achieved remission after 10% weight loss progressed to the 15% stage, and this produced no further remissions. (**b**) The weight loss at which HbA_1c_ of < 48 mmol/mol (< 6.5%) was first achieved is shown for each individual, illustrating the wide range of personal fat thresholds. (**c**) Change of HbA_1c_ at each step. The further decrease between end of weight loss and 12 months is notable. (**d**) Hepatic triglyceride content decreased with stepwise weight loss and was maintained at 12 months (shown as percentage of liver volume). The median for the normoglycaemic control group is shown as a horizontal dotted line. (**e**) The excess de novo lipogenesis at baseline, with return to normal after weight loss, was tracked by measuring the plasma concentration of triglyceride molecules with two palmitate fatty acid chains (50 carbon atoms but only one double bond). (**f**) Insulin secretion, calculated as disposition index following a standard test meal, increased with weight-loss-induced remission apart from at the 15% weight loss point when no remissions were seen. Figures are adapted from [67] under the terms of the CC BY Attribution License 4.0 (http://creativecommons.org/licenses/by/4.0/). TG, triglyceride. This figure is available as part of a downloadable slideset

The ReTUNE study (Reversal of Type 2 diabetes Upon Normalisation of Energy intake in the non-obese) specifically tested whether an individual threshold could be detected at which accumulation of fat in liver and pancreas caused diabetes [67]. As data from people with BMI ≥ 27 kg/m^2^ had already been collated, the study group involved people with BMI 21–27 kg/m^2^. It was important to identify the predicted threshold, and this was done by inducing weight loss in three 5% decrements in people with type 2 diabetes (Fig. 5a). Each decrement was achieved within 2 weeks and was followed by 6 weeks of weight stability. If correct, slim people should achieve remission at a weight loss that crossed their individual threshold.

The range of weight loss thresholds was shown to be wide (5.5–10.2%), with a median of 6.5% (Fig. 5b). Critically, the same pathophysiological mechanisms (Fig. 5c, d) were observed as in heavier people. At baseline, liver fat was raised by a similar threefold excess compared with weight-matched normoglycaemic control individuals and decreased to normal with weight loss. Fasting plasma insulin, plasma triglyceride and intrapancreatic fat were all high at baseline and returned to normal during the intervention. To test the concept that the increased plasma triglyceride was related to increased de novo lipogenesis, its fatty acid composition was examined. The levels of molecules with two palmitate fatty acid chains, an indicator of the rate of de novo lipogenesis, were increased at baseline but normalised with weight loss (Fig. 5e). The insulin response to a standard test meal was subnormal as expected at baseline and improved significantly (Fig. 5f).

From a practical clinical perspective, longer-term outcomes were striking. At 1 year after the rapid weight loss, 70% of the group had maintained non-diabetic glucose levels. There was no mean weight gain, unlike that seen with heavier people, from the end of the weight loss phases (mainly after 10% decrease) to the 12 month follow-up visit (Fig. 5a). Progression to the 15% weight loss step depended upon not having achieved remission and few participants undertook this. Weight stability was achieved with less-frequent follow-up than in DiRECT in which ~ 4 kg gain was seen over a comparable period [46]. It appears that people who start out being only moderately overweight find it easier to avoid weight regain. They have never been advised to lose weight before, in contrast to heavier people.

In ReTUNE it was imperative to study a group with a correct diagnosis as far as could be determined by specific tests to exclude monogenic diabetes and slow-onset type 1 diabetes. Both conditions were identified more frequently in the normal-weight group recruited than in the heavier wider population of people diagnosed with type 2 diabetes. The test results were not immediately available, so all initially recruited participants underwent weight loss. In two people with glucokinase deficiency this produced no change in HbA_1c_, and in two people with slow-onset type 1 diabetes a modest, temporary improvement was observed. Importantly, dietary weight loss in people with non-type 2 diabetes brought about no ill effects, as indeed seen with improved survival in type 1 diabetes on the very-low-energy Allen diet in the pre-insulin era [68]. At the first consultation for probable type 2 diabetes, there should be discussion of the uncertainties around the diagnosis of the type of diabetes. The clinical course after substantial weight loss can provide useful diagnostic clues.

The widely quoted upper level of ‘normal’ for liver fat content is based upon data from the population of the Dallas Heart Study; however, the participants had a mean BMI of 30 kg/m^2^ [69] and many would have had obesity-related fatty liver disease. In the non-diabetic control group of ReTUNE the median liver fat content was 1.3% (Fig. 5) and in a separate study the 95% limit of normal was found to be < 2% [70]. The application of inappropriate norms has led to the false assumption that only around 70% of people with type 2 diabetes have excess liver fat [71, 72]. The assumption of normal below a level of 5.5% may work well with respect to liver fibrosis or cirrhosis but not for metabolic dysfunction-associated steatotic liver disease.

Although other subtypes of diabetes may be identified in future, it is likely that new subtypes will be rare, given that 9/10 people with short-duration type 2 diabetes achieve remission with weight loss [43, 73].

## Summarising the research journey

The original destination of this experimental medicine odyssey was to identify the cause of type 2 diabetes and now this can simply be stated. If a person develops type 2 diabetes, they have accumulated more fat than can be tolerated by their own genetic constitution.

**Fig. 6:**
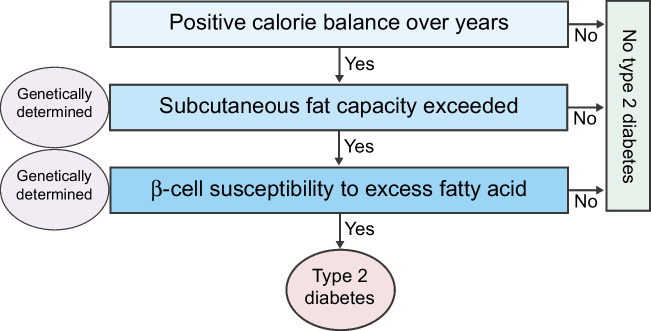
Summary of the hypothesis-driven pathophysiological and clinical studies. Taken together, the studies indicate that type 2 diabetes is caused by the single modifiable factor of positive energy balance over a long period. However, individuals are genetically heterogenous and two major stop–go steps can be identified. The capacity of subcutaneous tissues for fat varies considerably and if this metabolically safe storage site is relatively replete then plasma triglyceride levels will increase and fat is stored in ectopic sites, including liver and pancreas. Susceptibility of beta cells to fatty acid-induced suppression also appears to be genetically determined. However, even if an individual is genetically susceptible to type 2 diabetes in both of these respects, the disease does not develop unless positive energy balance causes excess fat to build up. Conversely, lack of susceptibility in either respect prevents development of hyperglycaemia despite considerable weight gain. Figure is reproduced with permission from [74]. This figure is available as part of a downloadable slideset

There is a single environmental, modifiable cause (Fig. 6). Type 2 diabetes only develops if an individual has eaten more than they require over a long period. However, there are two important stop–go stages, both determined by the lottery of inheritance [74]. Diabetes appears only when fat can no longer be stored safely under the skin. This subcutaneous capacity is now known to be determined by 53 genetic loci [75]. Even if fat spills over into ectopic sites, diabetes will only occur if there is also genetic susceptibility to fat suppression at the level of the beta cell, as originally shown by Unger and colleagues [19–21]. Most people do not have beta cell susceptibility to fat: in people with BMI over 40 kg/m^2^, 73% do not have diabetes [76]. Even if full genetic susceptibility is present, the writing is clearly on the wall: no positive energy balance, no diabetes. This point is nicely underscored by Eliott Joslin’s observation of no type 2 diabetes in Pima Indians living as subsistence farmers, despite its very high prevalence in today’s environment [77, 78].

Claude Bernard set out the methods required to establish cause and effect in human health and disease. First, relevant normal function must be understood. Next, all sound pathophysiological information about the disease must be considered and a hypothesis postulated. Then, ‘stimulate a change’ in the system, the effect of which can be predicted by the hypothesis and repeat the physiological observations. Standing on the shoulders of giants, it has been possible to undertake a research journey with a clear conclusion. Type 2 diabetes is a condition of homogenous aetiology in genetically heterogenous individuals. It is a potentially reversible metabolic state and this understanding can be incorporated into clinical practice.

## Supplementary Information

Below is the link to the electronic supplementary material.

Slideset of figures (PPTX 2.24 MB)

## Abbreviations

Counterbalance: COUNTERacting BetA cell failure by Long term Action to Normalise Calorie intakE
Counterpoint: COUNTERacting Pancreatic inhibitiOn of INsulin secretion by Triglyceride
NHS: National Health Service
ReTUNE: Reversal of Type 2 diabetes Upon Normalisation of Energy intake in the non-obese
UKPDS: UK Prospective Diabetes Study
VLDL-TG: VLDL-triglyceride
