# Supplementary material for: Aetiology of type 2 diabetes: an experimental medicine odyssey
Source: Diabetologia. 2025 May 2;68(7):1375–89. doi: 10.1007/s00125-025-06428-0 (PMC12176950; doi:10.1007/s00125-025-06428-0)
Supplement: Supplementary file 1 — Slideset of figures (PPTX 2.24 MB) [file 125_2025_6428_MOESM1_ESM.pptx]

## Slide 1
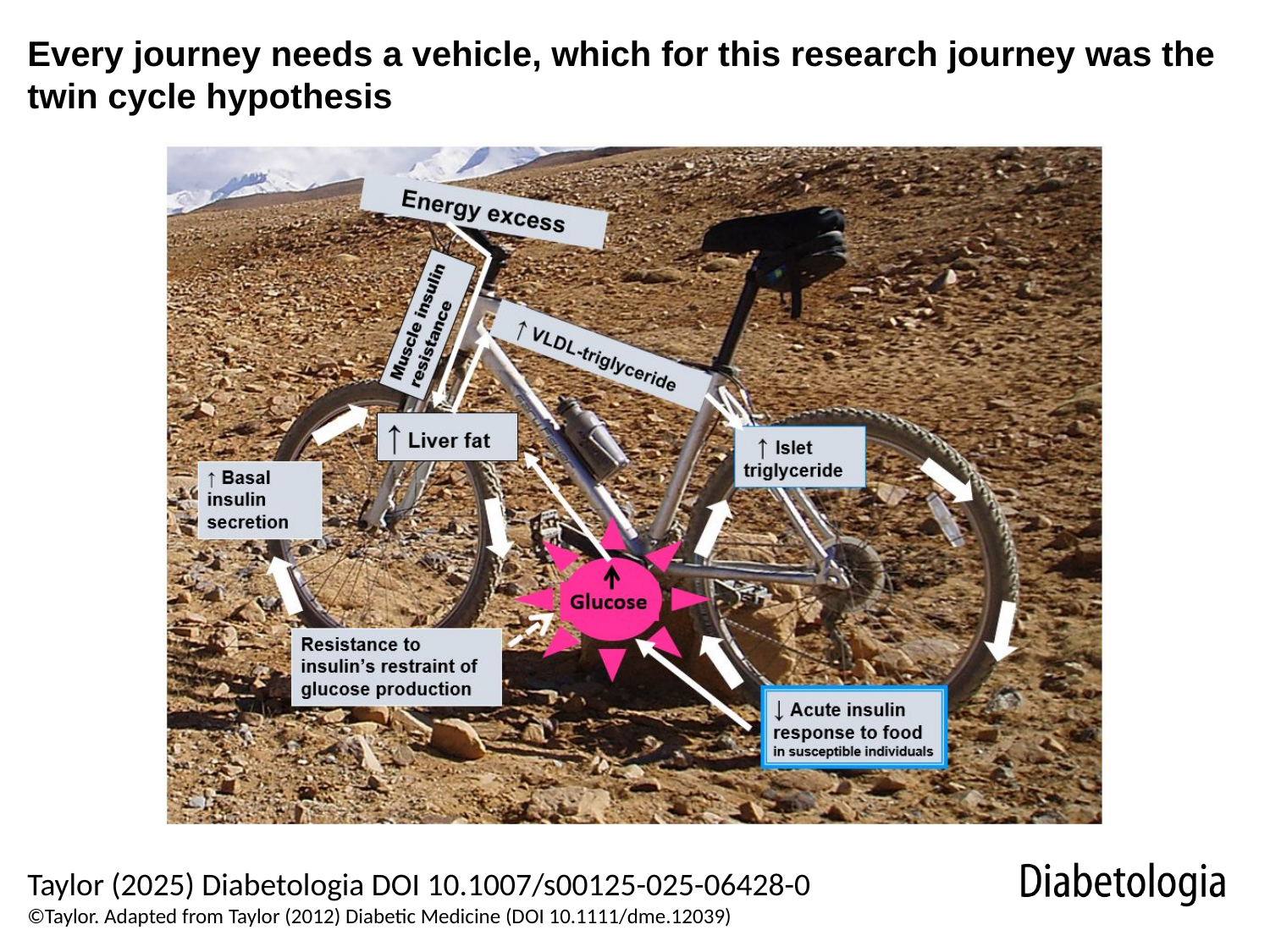

Every journey needs a vehicle, which for this research journey was the twin cycle hypothesis
Taylor (2025) Diabetologia DOI 10.1007/s00125-025-06428-0
©Taylor. Adapted from Taylor (2012) Diabetic Medicine (DOI 10.1111/dme.12039)

## Slide 2
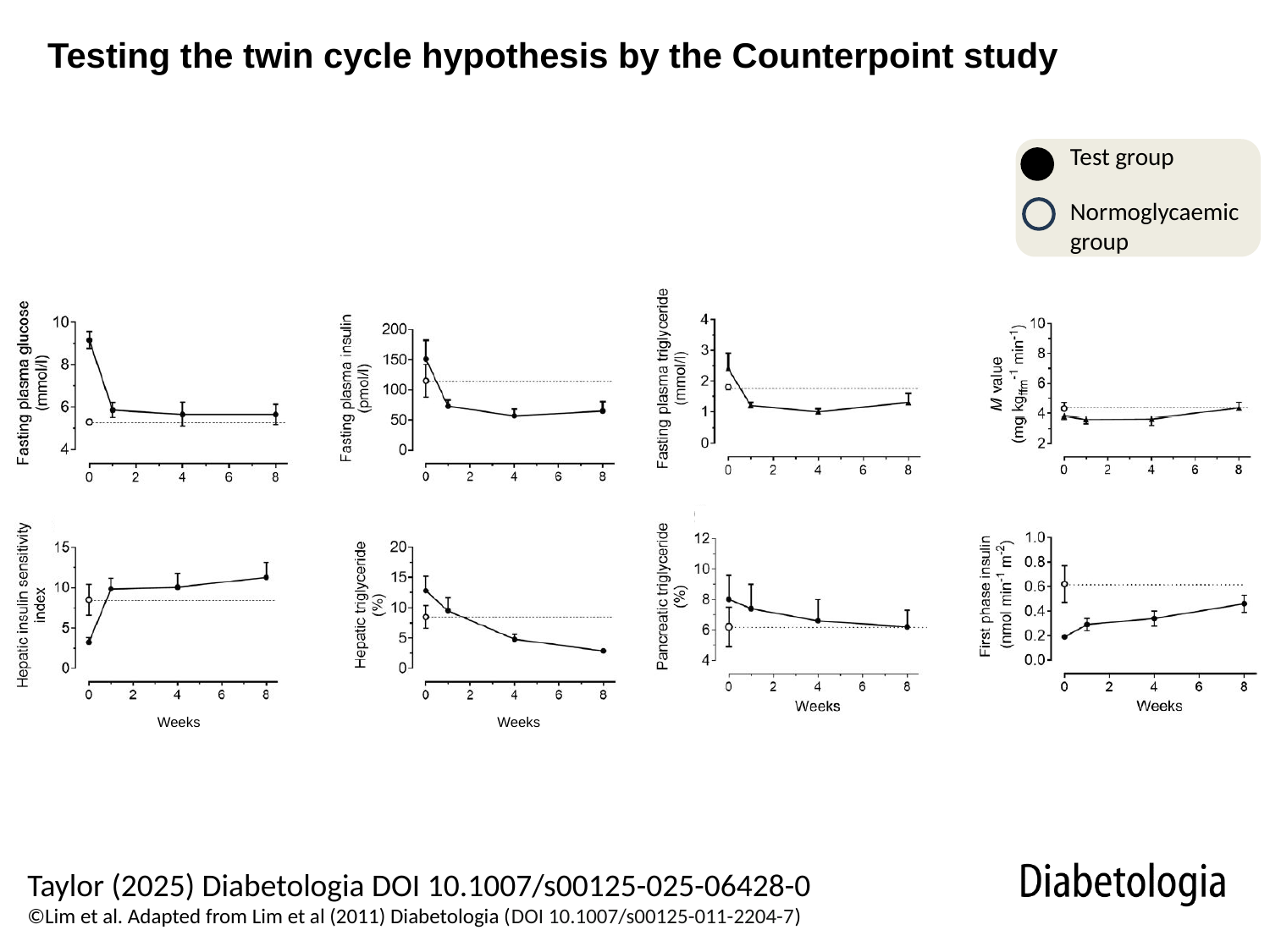

Testing the twin cycle hypothesis by the Counterpoint study
Test group
Normoglycaemic group
Weeks
Weeks
Taylor (2025) Diabetologia DOI 10.1007/s00125-025-06428-0
©Lim et al. Adapted from Lim et al (2011) Diabetologia (DOI 10.1007/s00125-011-2204-7)

## Slide 3
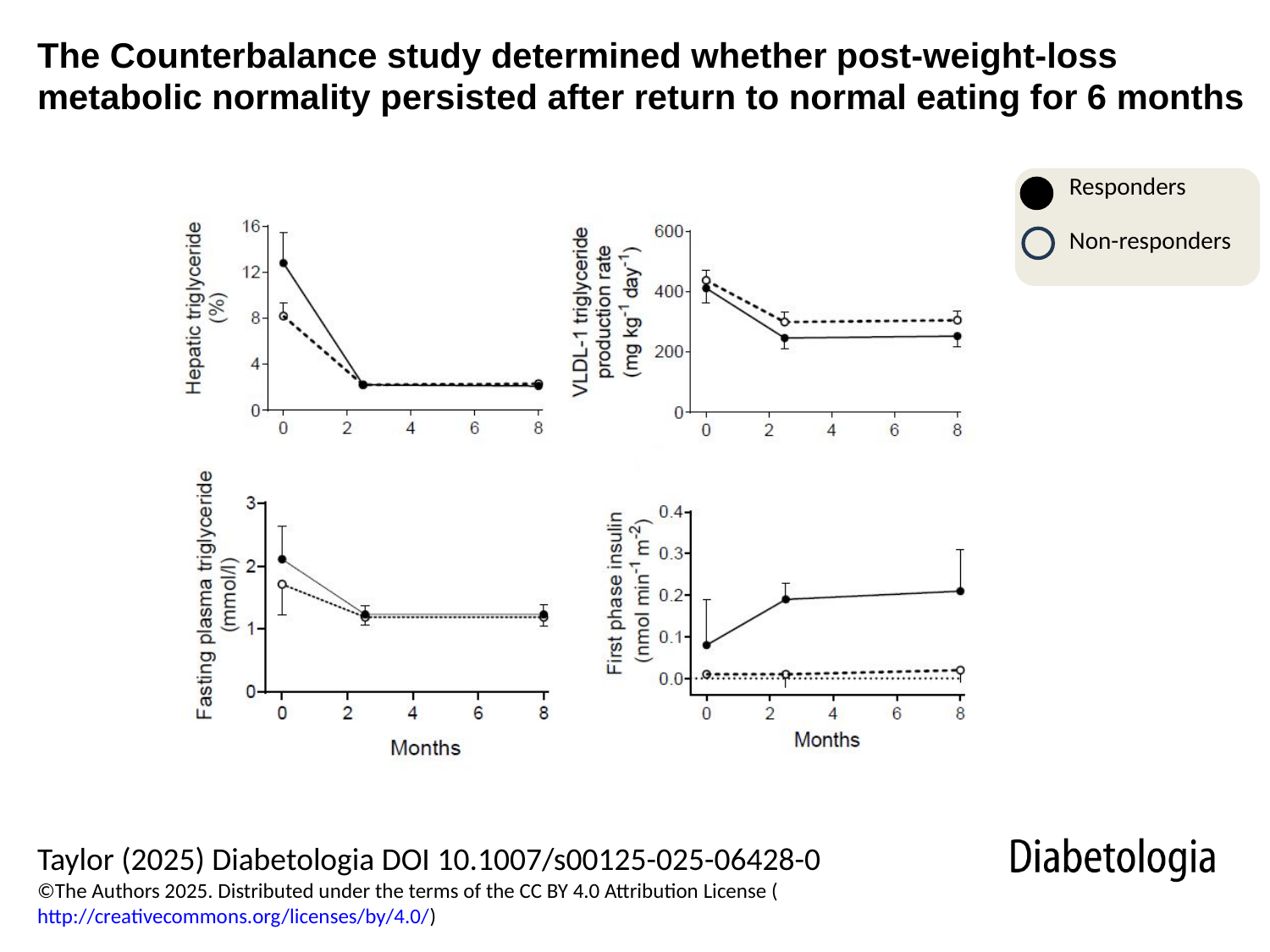

The Counterbalance study determined whether post-weight-loss metabolic normality persisted after return to normal eating for 6 months
Responders
Non-responders
Taylor (2025) Diabetologia DOI 10.1007/s00125-025-06428-0
©The Authors 2025. Distributed under the terms of the CC BY 4.0 Attribution License (http://creativecommons.org/licenses/by/4.0/)

## Slide 4
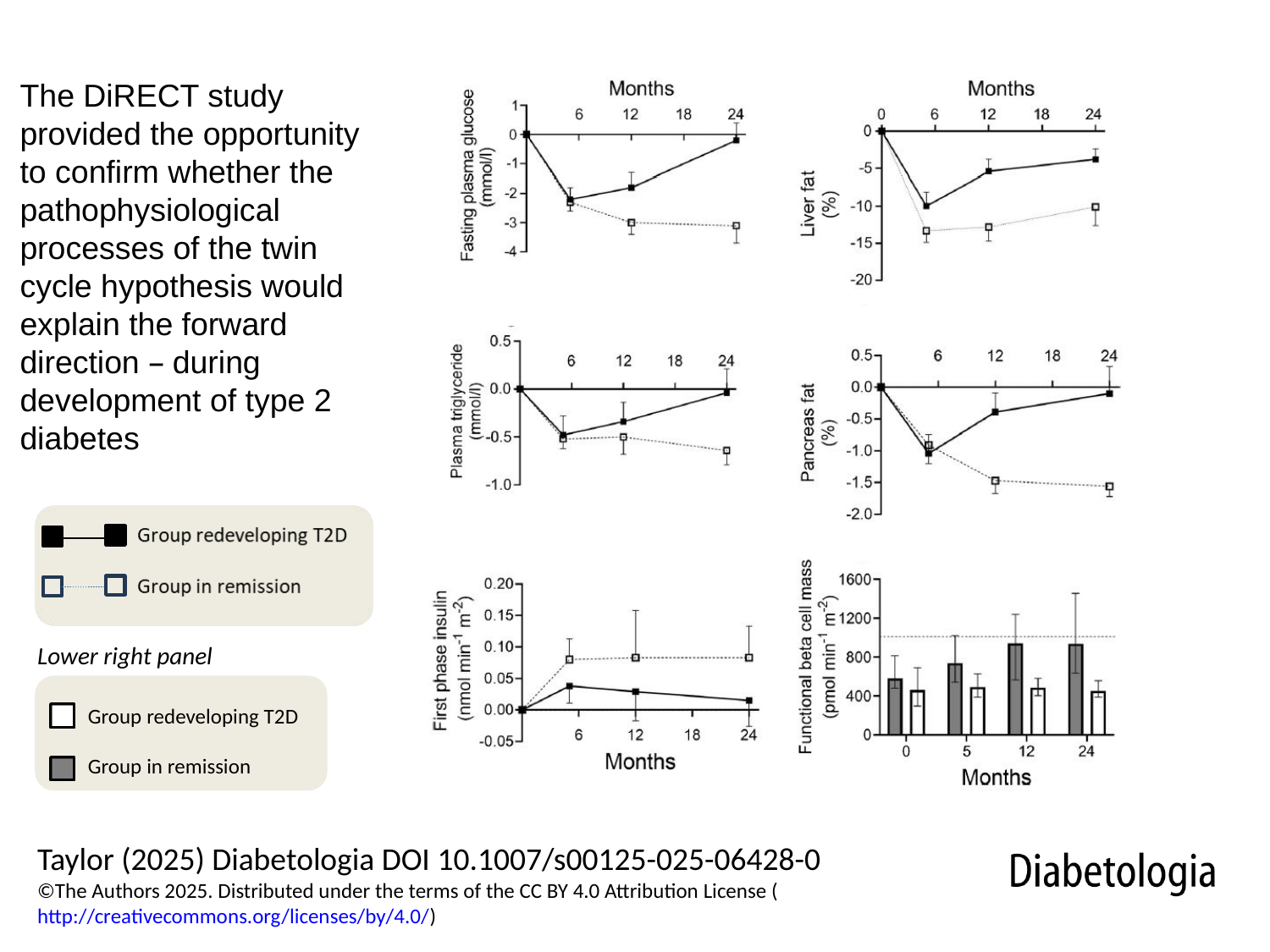

The DiRECT study provided the opportunity to confirm whether the pathophysiological processes of the twin cycle hypothesis would explain the forward direction – during development of type 2 diabetes
Lower right panel
Group redeveloping T2D
Group in remission
Taylor (2025) Diabetologia DOI 10.1007/s00125-025-06428-0
©The Authors 2025. Distributed under the terms of the CC BY 4.0 Attribution License (http://creativecommons.org/licenses/by/4.0/)

## Slide 5
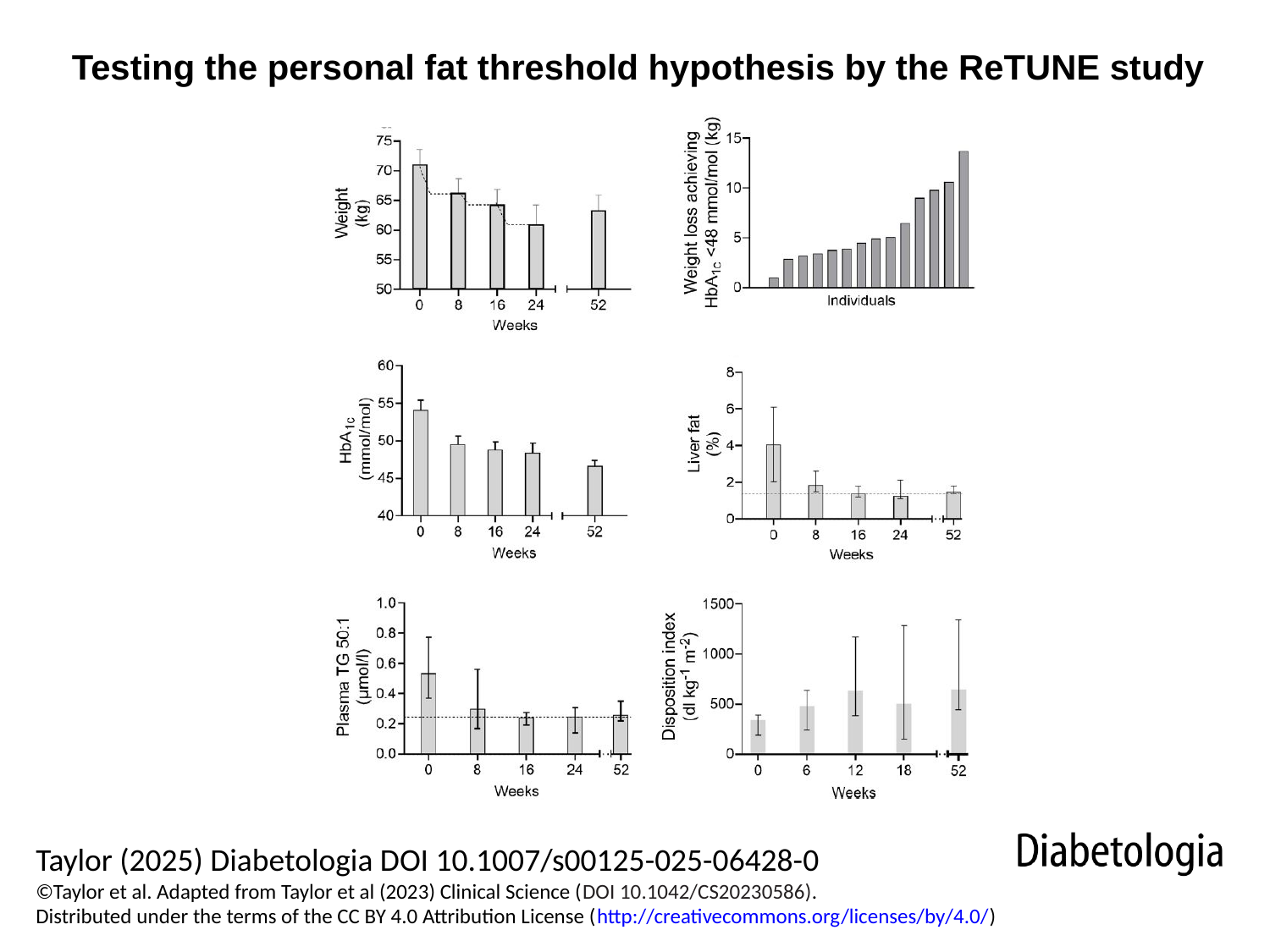

# Testing the personal fat threshold hypothesis by the ReTUNE study
Taylor (2025) Diabetologia DOI 10.1007/s00125-025-06428-0
©Taylor et al. Adapted from Taylor et al (2023) Clinical Science (DOI 10.1042/CS20230586).
Distributed under the terms of the CC BY 4.0 Attribution License (http://creativecommons.org/licenses/by/4.0/)

## Slide 6
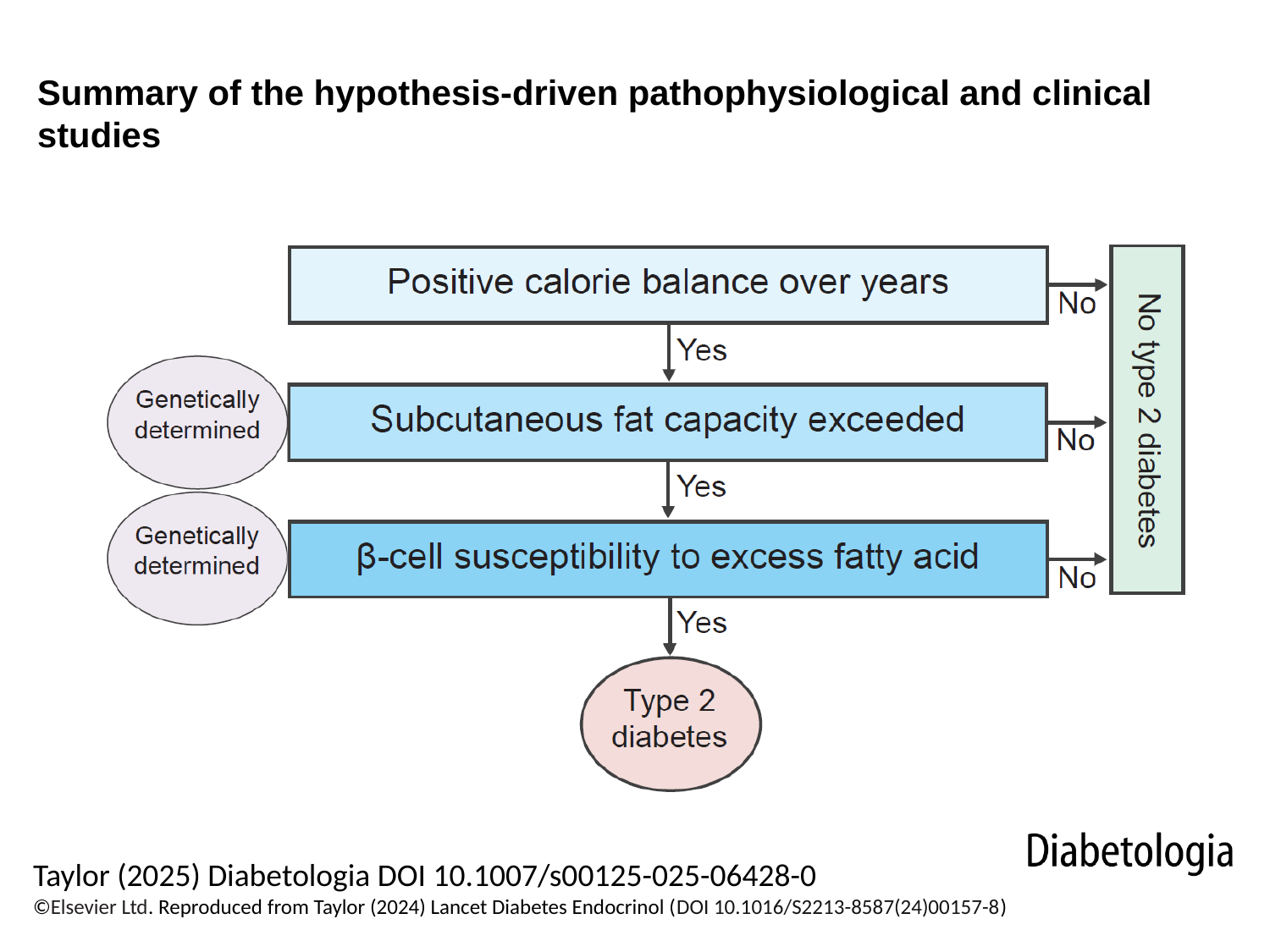

Summary of the hypothesis-driven pathophysiological and clinical studies
Taylor (2025) Diabetologia DOI 10.1007/s00125-025-06428-0
©Elsevier Ltd. Reproduced from Taylor (2024) Lancet Diabetes Endocrinol (DOI 10.1016/S2213-8587(24)00157-8)
